# Mucosal Adjuvanticity of Fibronectin-Binding Peptide (FBP) Fused with *Echinococcus multilocularis* Tetraspanin 3: Systemic and Local Antibody Responses

**DOI:** 10.1371/journal.pntd.0001842

**Published:** 2012-09-27

**Authors:** Zhisheng Dang, Jinchao Feng, Kinpei Yagi, Chihiro Sugimoto, Wei Li, Yuzaburo Oku

**Affiliations:** 1 College of Life and Environmental Sciences, Minzu University of China, Beijing, People's Republic of China; 2 Parasitology Laboratory, School of Veterinary Medicine, Faculty of Agriculture, Tottori University, Tottori, Japan; 3 Department of Biological Science, Hokkaido Institute of Public Health, Sapporo, Japan; 4 Division of Collaboration and Education, Research Center for Zoonosis Control, Hokkaido University, Sapporo, Japan; 5 Qinghai Academy of Animal and Veterinary Medicine, Qinghai, People's Republic of China; The George Washington University Medical Center, United States of America

## Abstract

**Background:**

Studies have shown that a bacterial fibronectin attachment protein (FAP) is able to stimulate strong systemic and mucosal antibody responses when it is used alone or co-administrated with other antigens (Ags). Thus, it has been suggested to be a promising adjuvant candidate for the development of efficient vaccines. However, the co-administered Ags and FAP were cloned, expressed and purified individually to date. In a recent study, we first evaluated the adjuvanticity of a fibronectin-binding peptide (FBP, 24 amino acids) of *Mycobacterium avium* FAP fused with *Echinococcus multilocularis* tetraspanin 3 (Em-TSP3) by detecting systemic and local antibody responses in intranasally (i.n.) immunized BALB/c mice.

**Methodology/Principal Findings:**

Em-TSP3 and FBP fragments were linked with a GSGGSG linker and expressed as a single fusion protein (Em-TSP3-FBP) using the pBAD/Thio-TOPO expression vector. BALB/c mice were immunized i.n. with recombinant Em-TSP3-FBP (rEm-TSP3-FBP) and rEm-TSP3+CpG and the systemic and local antibody responses were detected by ELISA. The results showed that both rEm-TSP3-FBP and rEm-TSP3+CpG evoked strong serum IgG (*p*<0.001) and IgG1 responses (*p*<0.001), whereas only the latter induced a high level IgG2α production (*p*<0.001), compared to that of rEm-TSP3 alone without any adjuvant. There were no significant differences in IgG and IgG1 production between the groups. Low level of serum IgA and IgM were detected in both groups. The tendency of Th1 and Th2 cell immune responses were assessed via detecting the IgG1/IgG2α ratio after the second and third immunizations. The results indicated that i.n. immunization with rEm-TSP3-FBP resulted in an increased IgG1/IgG2α ratio (a Th2 tendency), while rEm-TSP3+CpG caused a rapid Th1 response that later shifted to a Th2 response. Immunization with rEm-TSP3-FBP provoked significantly stronger IgA antibody responses in intestine (*p*<0.05), lung (*p*<0.001) and spleen (*p*<0.001) compared to those by rEm-TSP3+CpG. Significantly high level IgA antibodies were detected in nasal cavity (*p*<0.05) and liver (*p*<0.05) samples from both groups when compared to rEm-TSP3 alone without any adjuvant, with no significant difference between them.

**Conclusions:**

I.n. administration of rEm-TSP3-FBP can induce strong systemic and mucosal antibody responses in immunized BALB/c mice, suggesting that fusion of Em-TSP3 with FBP is a novel, prospective strategy for developing safe and efficient human mucosal vaccines against alveolar echinococcosis (AE).

## Author Summary

*Echinococcus* metacestodes form a laminated layer and develop strategies to escape host immune responses once the infection established on the liver of intermediated host. One of the most important strategies is thought to be immunoregulation, where some molecules (e.g., antigen B) impair dendritic cell (DC) differentiation and polarize immature DC maturation towards a non-protective Th2 cell response. Therefore, it is more feasible to kill *Echinococcus* oncospheres in the early stage of infection in the intestine and blood. Systemic and local immune responses are believed to play a crucial role on oncosphere exclusion. Among antigen delivery systems, i.n. administration is the most efficient one, inducing both systemic and a full-range of mucosal immune responses. FAP is necessary to *M. avium* and *S. pyogenes* to efficiently attach and invade epithelial cells, and has been suggested as a potent vaccine adjuvant. Mucosal immune responses are induced after FAP binds to the fibronectin protein of host microfold (M) cells and DCs are activated. We developed a one-step delivery system where FAP and other Ags can be expressed, purified and immunized as one protein. The systemic and, in particular, the mucosal antibody responses induced by the fusion protein were detected to evaluate the adjuvanticity of FBP.

## Introduction

*Echinococcus multilocularis* infection in humans and rodents occurs after oncosphere-containing eggs are orally ingested. Oncospheres penetrate the mucosa of the small intestine and migrate via the hepatic vein to the liver where they form cyst masses and increasingly transform into multiple vesicles filled with fluid and protoscoleces. Only oncospheres hatching from eggs in the small intestine are able to transit the mucosa. Therefore, an effective echinococcosis vaccine must stimulate a local mucosal response to block both infection and disease development, as is the case for many micropathogens [1]. In addition, a systemic response is necessary to achieve protection against the spread of oncospheres. Parent vaccines are generally ineffective in stimulating mucosal immunity, whereas mucosally delivered immunogens trigger both local and systemic immune responses [1], [2]. Administration of Ags with potent mucosal adjuvants is used to ensure that an efficient immune response is elicited. To data, only a few molecules have shown their potentials as mucosal adjuvants. However, their toxicity and potential side effects limited their use in human vaccination [3]–[7]. CpG oligodeoxynucleotides (ODN) has been proved to be an ideal mucosal adjuvant due to its non-toxicity and ability to induce strong systemic and/or local immune responses [8]–[12]. We recently showed that both systemic and local antibody responses were stimulated when CpG ODN was co-administered with rEm-TSP3 to BALB/c mice intranasally (i.n.). Unfortunately, they failed to induce a satisfied intestinal IgA response [13]; thus, this study focuses to find out other molecules as an adjuvant which may enhance intestinal IgA immune response.

Studies showed that the fibronectin-binding protein of *S. pyogenes* (SfbI) stimulates efficient, long-lasting serum and mucosal antibody responses against SfbI or other co-administered model Ags such as ovalbuin (OVA) and beta-galactosidase (beta-gal) [14]–[17]. The fibronectin-binding/attachment proteins of *S. pyogenes* (SfbI) and *M. avium* (FAP) are necessary for efficient attachment and invasion of epithelial cells by these microorganisms. After SfbI/FAP binds to the fibronectin protein on the surface of host M cells, DCs are activated and induce mucosal immune responses [18]–[20]. However, the use of FAP as an adjuvant for co-administration with other protective Ags requires separate cloning, expression and purification of each protein. To overcome this problem and develop a one-step delivery system, we cloned the linked fibronectin-binding peptide (FBP) of *M. avium* FAP and Em-TSP3 into a pBAD/Thio-TOPO expression plasmid. The identification of short FBP (72 bp) greatly facilitated this work [19], [21], [22], because it is easy to synthesize. In this study, the adjuvanticity of the fusion form of FBP and Em-TSP3 (rEm-TSP3-FBP) was evaluated by detecting systemic and mucosal antibody responses against Em-TSP3 vaccine.

## Materials and Methods

### Ethics statement

This study was carried out in strict accordance to the recommendations set out in the Guidelines for Animal Experimentation of the Japanese Association for Laboratory Animal Science and the protocol for the animal experiments was approved by the ethics committee of Hokkaido University (Permit Number: 09-0144) and the Hokkaido Institute of Public Health (Permit Number: K20-6). I.n. immunization and sacrification of mice were performed under isoflurane anesthesia and all efforts were made to minimize suffering.

### Experimental animals

Fifteen five-week-old BALB/c mice (male) were divided into 5 groups and maintained in cages in a P3 animal room at 23–25°C with a 12 h light/dark cycle. Litter was cleaned weekly. They were provided with food and water *ad libitum*. Mice were immunized at 6 weeks of age.

### Recombinant plasmid constructions

**Figure 1:**
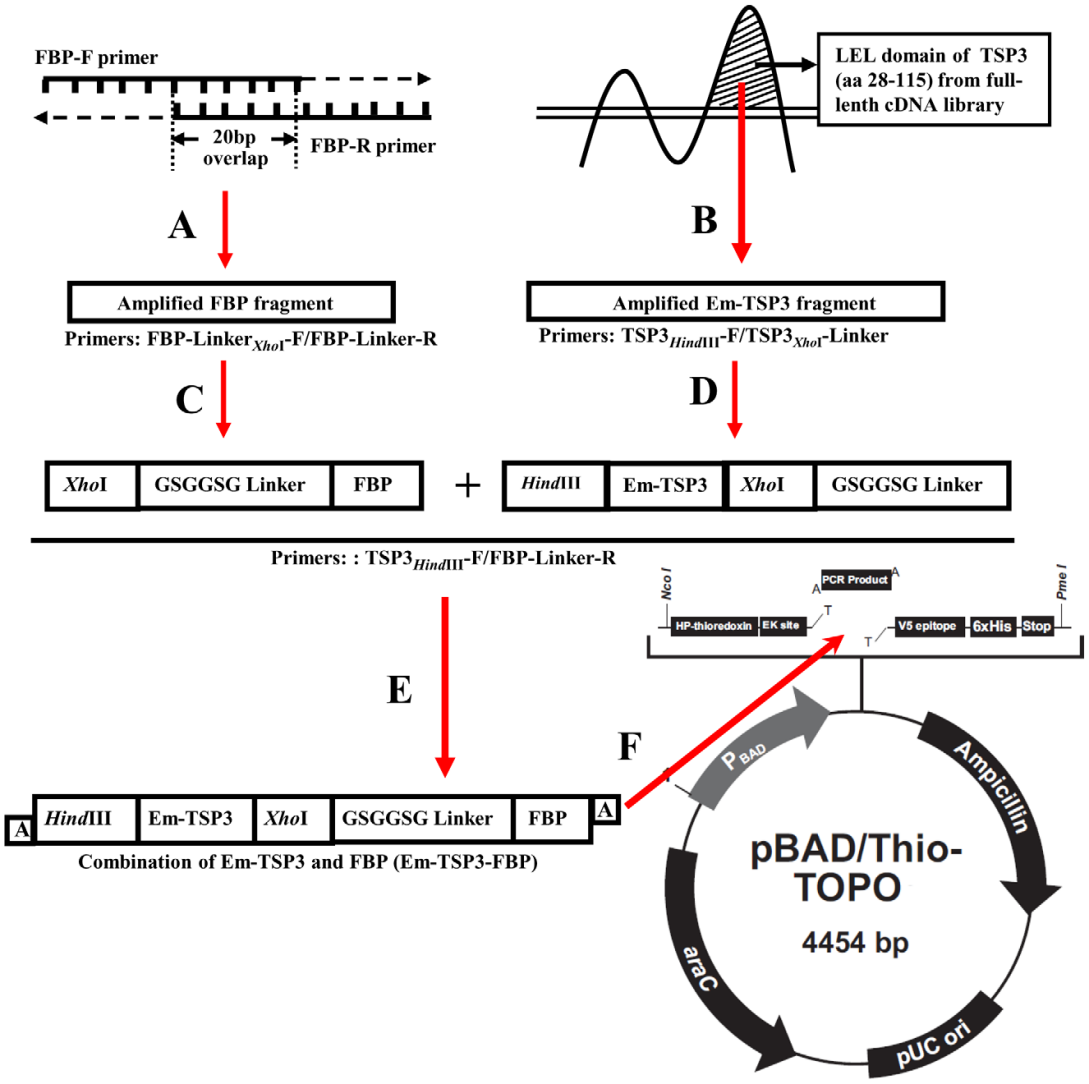
Schematic illustration of construction of fusion rEm-TSP3-FBP plasmid. (A) Synthesis of FBP. (B) Amplification of Em-TSP3 LEL from full-length cDNA library. (C) Ligation of linker to FBP fragment. (D) Ligation of linker to Em-TSP3 fragment. (E) Combination of FBP and Em-TSP3 (Em-TSP3-FBP) by fusion PCR. (F) Insertion of Em-TSP3-FBP into pBAD/Thio-TOPO cloning and expression vector.

Em-TSP3-FAP recombinant plasmid construction was performed as previously described [23] and illustrated in Figure 1. The primers used for amplification of fragments were listed in Table 1. Briefly, FBP (peptide 265–288 of *M. avium paratuberculosis* FAP) was amplified by PCR with FBP-F/FBP-R primers and an *Xho*I restriction site and GSGGSG linker (nucleotide sequence: GGTAGCGGCGGTTCTGG T) introduced into FBP fragment by PCR with FBP-Linker*_Xho_*_I_-F/FBP-Linker-R primers. The region encoding the LEL (larger extracellular loop) domain of Em-TSP3 was amplified from the full-length enriched cDNA library of *E. multilocularis* larvae. The GSGGSG linker, *Hind*III and *Xho*I restriction sites were introduced into Em-TSP3 fragment by PCR with TSP3*_Hind_*_III_-F/TSP3*_Xho_*_I_-Linker-R primers. These two reconstructed fragments were combined by fusion PCR with TSP3*_Hind_*_III_-F/FBP-Linker-R primers. The combined Em-TSP3-FBP fragment was then subcloned into pBAD/Thio-TOPO expression vector (Invitrogen, USA). Gene TaqNT polymerase (Nippon Gene, Japan) was used in PCR reaction.

**Table 1:** List of primers used for fragments amplification.

Fragment (bp)	Primers	Sequence of primers	Tm (°C)
**FBP (72)**	FBP-F	5′GGCAACGCGCAACGCTGGTTCGTCGTCTGGCTGGGCACCTCGAACG3′	60
	FBP-R	5′CTTGGCCGCGACCTTGTCCACCGGGTCGTTCGAGGTGCCCAGCCAG3′	
**FBP-Linker (96)**	FBP-Linker*_Xho_*_I_-F	5′*CTCGAG*GGTAGCGGCGGTTCTGGTGGCAACGCGCAACGCTGG3′	60
	FBP-Linker-R	5′TTACTTGGCCGCGACCTTGTCCA3′	
**TSP3-Linker (294)**	TSP3*_Hind_*_III_-F	5′*AAGCTT*ATTCCTGATAACCTAAACAAAGCAG3′	55
	TSP*_Xho_*_I_-linker-R	5′GAACCGCCGCTACC*CTCGAG*GAGG GTTTTGTTCTCTGCCA3′	
**TSP3-FBP (366)**	TSP3*_Hind_*_III_-F	5′*AAGCTT*ATTCCTGATAACCTAAACAAAGCAG3′	60
	FBP-Linker-R	5′TTACTTGGCCGCGACCTTGTCCA3′	

F = forward;

R = reverse;

Tm = melting temperature.

### Protein expression and purification

Recombinant protein expression and purification was performed as previously described [23]. Briefly, *Escherichia coli* TOP10 cells (Invitrogen, USA) were transformed with recombinant plasmid according to the manufacturer's instructions (pBAD/TOPO-ThioFusion Expression Kit, Invitrogen, USA). Recombinant protein from *E. coli* lysates was purified with a HisTrap affinity column (HisTrap FF crude 1 ml, GE Healthcare, USA) under non-denaturing conditions and stored at −80°C. rEm-TSP3 alone was also expressed, purified and used as the antigen in ELISA instead of rEm-TSP3-FBP, to deplete the reaction caused by FBP peptide.

### Hydrophobicity plot prediction and tertiary structure analysis of expressed proteins

To confirm the solubility of rEm-TSP pre- and post-fusion with FBP, hydrophobicity plot was predicted by the Kyte–Doolittle hydropathy plot program (http://fasta.bioch.virginia.edu/fasta_www2/fasta_www.cgi?rm=misc1). The amino acid sequence of Em-TSP3 was aligned with protein sequences in the structural database using the Phyre (Protein Homology/analogy Recognition Engine) server at Imperial College (http://www.sbg.bio.ic.ac.uk/phyre/) [24] and the 3-dimensional structure was further activated.

### Immunization of mice and sample collection

BALB/c mice (3 per group) were immunized i.n. three times with PBS, PBS+CpG, rEm-TSP3, rEm-TSP3+CpG and rEm-TSP3-FBP on a weekly basis. A dose of 50 µg per mouse (in 50 µl PBS) was used for three immunizations. This is independently reported here because this work was focused on evaluating the adjuvanticity of FBP as a novel strategy for vaccine development. CpG OND data is cited here to show the adjuvanticity of FBP under the same experiment conditions [13]. A dose of 1 nM CpG OND (Hokkaido System Science, Japan) was used per mouse. Retro-orbital blood collection was performed on mice one week after the second and third immunizations using glass capillary pipettes (Hirschmann, Germany) and the serum was isolated. Mice from each group were sacrificed one week after the third immunization and the nasal cavity washes, intestine, liver, lung and spleen were collected in 500 µl of PBS (pH 7.4). A 10-cm fragment of the ileal region was excised and the intestinal tube was opened and immersed in 250 µl of PBS; liver, lung and spleen were homogenized in 500 µl of PBS separately and vigorously vortexed, followed by centrifugation to remove insoluble debris. The sera and collected supernatants were stored at −20°C for further use.

### Detection of systemic and local antibody responses by ELISA

Indirect ELISA was conducted for the antibody analysis as previously described [23]. Briefly, 96-well microtiter plates (Corning, USA) were coated with rEm-TSP3 protein (0.25 µg/well), blocked with 5% skim milk. For serum IgG, IgG1, IgG2α, IgA and IgM detection, plates were incubated with sera at a dilution of 1∶ 2,000 followed by incubation with horseradish peroxidase (HRP)-conjugated anti-mouse IgG (Invitrogen, USA), IgG1 (Rockland, USA), IgG2α (Southern Biotech, USA), IgA (Invitrogen, USA) and IgM (MP Biomedicals, USA). For IgA antibody detection in nasal cavity, intestine and liver, plate were incubated with nasal cavity washes, intestine washes, liver, lung and spleen extracts at a dilution of 1∶ 10, respectively, followed by incubation with HRP-conjugated anti-mouse IgA. A color reaction was developed by the addition of 100 µl of TMB (3, 3′, 5, 5′-tetramethylbenzidine) substrate (Dojindo, Japan). Absorbance was measured at 450 nm on a Biotrak II plate reader (Amersham Biosciences, USA).

### Statistical analyses

Data was analyzed using one-way ANOVA followed by a multiple comparison Tukey's test. Differences were considered statistically significant at *p*<0.05, very significant at *p*<0.01 and extremely significant at *p*<0.001.

## Results

### Amplification of Em-TSP3-FBP fragment by fusion PCR

**Figure 2:**
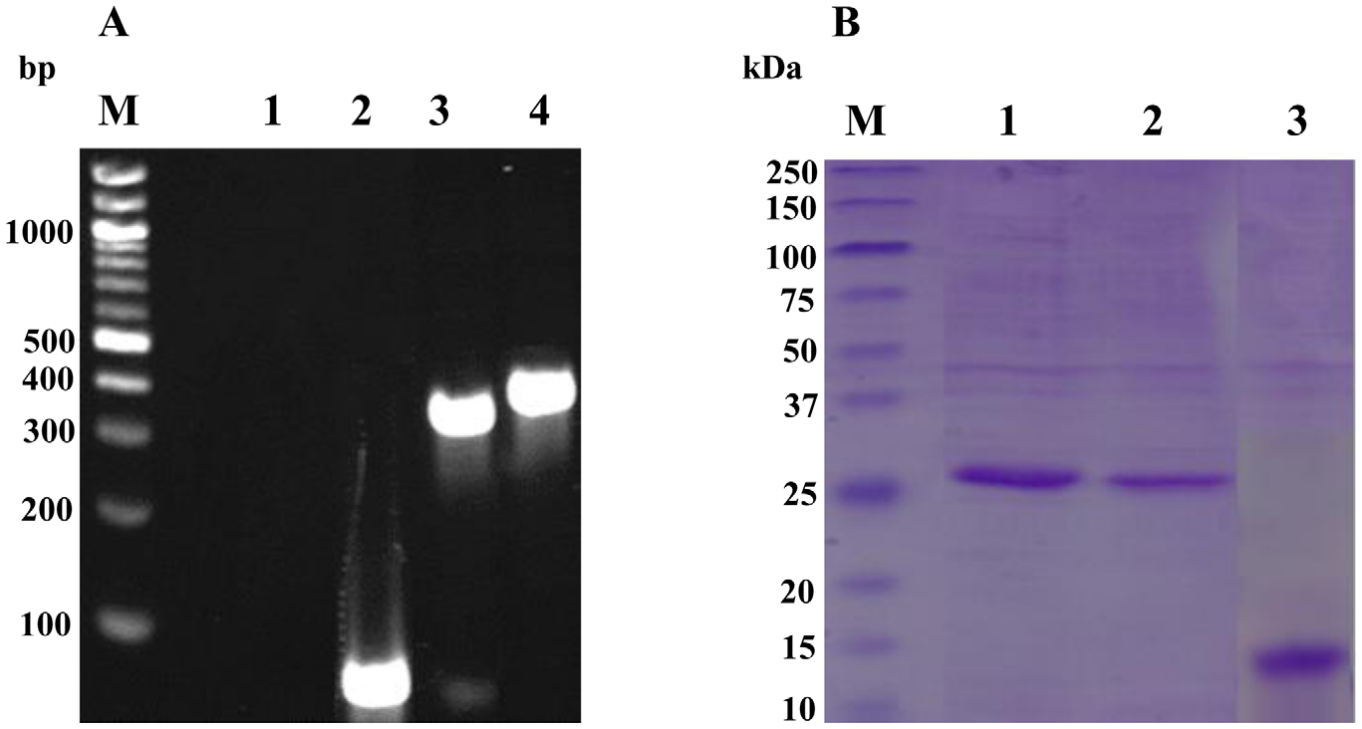
Cloning and expression of recombinant Em-TSP3-FBP protein. (A) Synthesis of FBP and Cloning of the LEL and Em-TSP3-FBP. M, marker. Lane 1. Negative control (H_2_O). Lane 2. Synthesized FBP (72 bp). Lane 3. PCR product of Em-TSP3 LEL (276 bp). Lane 4. PCR product of Em-TSP3-FBP (366 bp). (B) Expression of recombinant Em-TSP3-FBP (fused with thioredoxin (TRX)). SDS-PAGE gel stained with Coomassie Blue showing purified rEm-TSP3 (Lane 1, 26.2 kDa), Em-TSP3-FBP (Lane 2, 29.3 kDa) and control TRX (Lane 3, 16 kDa). M, the molecular weight marker.

Em-TSP3 (276 bp including *Xho*I, *Hind*III sites) and FBP (72 bp) were amplified separately and linked with GSGGSG linker (18 bp) by fusion PCR. A band of 366 bp was observed in the agarose gel under ultraviolet light (Figure 2A).

### Expression of fusion rEm-TSP3-FBP protein

SDS-PAGE analysis showed that Em-TSP3 at approximately 26 kDa of and Em-TSP3-FBP at 29 kDa were expressed as predicted (Figure 2B).

### Solubility and 3-D structure of expressed proteins

**Figure 3:**
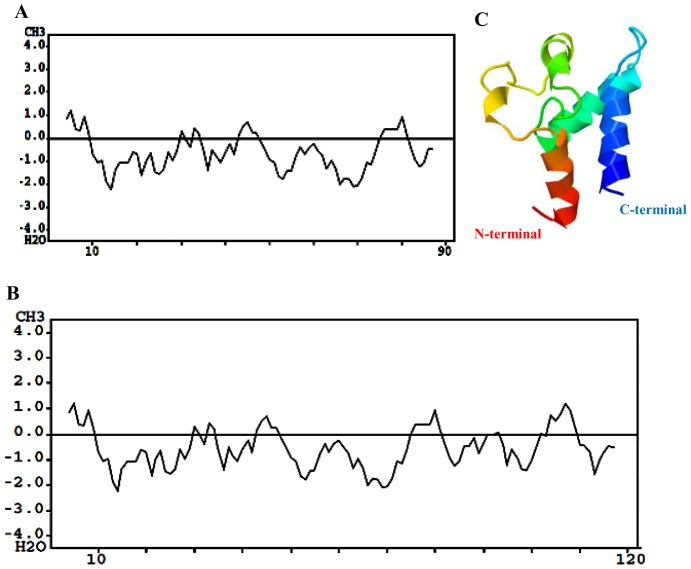
Solubility and 3-D structure of expressed proteins. Comparison of hydropathy plots of Em-TSP3 (A) and Em-TSP3-FBP (B). Hydropathy plots of the predicted amino acid sequences were obtained using the Kyte-Doolittle hydropathy plot program (http://fasta.bioch.virginia.edu/fasta_www2/fasta_www.cgi?rm=misc1). Hydrophobic residues are positive. (C) 3-D structure prediction of Em-TSP3 LEL domain. The amino acid sequence of Em-TSP3 (LEL) was aligned with protein sequences in the structural database using the Phyre (Protein Homology/analogy Recognition Engine) server at Imperial College (http://www.sbg.bio.ic.ac.uk/phyre/). The three-dimensional structure was further activated.

The hydrophobicity plot prediction showed that after the fusion of Em-TSP3 and FBP, there was no significant change in solubility compared to Em-TSP3 alone (Figure 3A,3B). The 3-dimensional structure illustration of Em-TSP3 showed that the C-terminal is exposed outside (Figure 3C).

### Serum antibody responses

**Figure 4:**
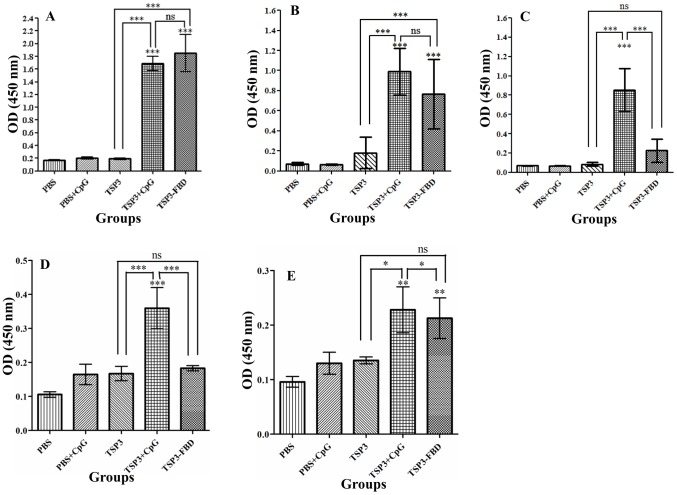
ELISA detection of serum antibody responses. Serum antibody responses were detected in BALB/c mice immunized with rEm-TSP3+CpG or rEm-TSP3-FBP intranasally after the third immunization. Absorbance (OD value at 450 nm) of sera IgG (A), IgG1 (B), IgG2α (C), IgM (D) and IgA (E) were presented in different bars. The S.D. is indicated by vertical lines. Significant differences between the vaccinated groups and the PBS control group are denoted by an asterisk over the bar. Significant differences between any of two groups are denoted by an asterisk over the line connecting them. n = 3 per group; *p<0.05 (significant); **p<0.01 (very significant); ***p<0.001 (extremely significant).

Systemic antibody responses against rEm-TSP3+CpG and rEm-TSP3-FBP evoked by i.n. administrations were detected by ELISA. Compared to PBS control or Em-TSP3 alone, significant serum IgG (*p*<0.001) (Figure 4A) and IgG1 (*p*<0.001) (Figure 4B) antibody responses were detected in both the rEm-TSP3+CpG and rEm-TSP3-FBP groups (*p*<0.001). Only the former protein induced a significantly higher IgG2α response (*p*<0.001) (Figure 4C). No significant differences were observed between these two groups in IgG and IgG1 production. Very low level serum IgM (Figure 4D) and IgA antibodies (Figure 4E) were detected, with a significant difference in IgM production (*p*<0.001) between the groups (Figure 4D). There were no significant serum antibody responses to rEm-TSP3 alone (Figure 4A–4D).

The Th1 and Th2 cell responses were assessed via IgG1/IgG2α ratio (Table 2). Two weeks post-immunization, a predominantly Th1 response was detected in mice immunized with rEm-TSP3+CpG, but the IgG1/IgG2α ratio dramatically reduced at 3 weeks post-immunization (a Th2 tendency). Conversely, immunization with rEm-TSP3-FBP resulted in a Th2-predominated response.

**Table 2:** Th1/Th2 tendency post-the third immunization with rEm-TSP3+CpG and rEm-TSP3-FBP.

Protein	Weeks p.i.	IgG1*	IgG2α*	IgG1/IgG2α ratio
**rEm-TSP3+CpG**	2 weeks p.i.	0.344±0.098	0.432±0.148	0.80
	3 weeks p.i.	1.014±0.233	0.834±0.201	1.22
**rEm-TSP3-FBP**	2 weeks p.i.	0.214±0.110	0.148±0.099	1.446
	3 weeks p.i.	0.667±0.347	0.225±0.108	2.964

i.n. = intranasal;

p.i. = post-immunization;

* Values are presented as means ± standard deviation.

### Mucosal antibody responses

**Figure 5:**
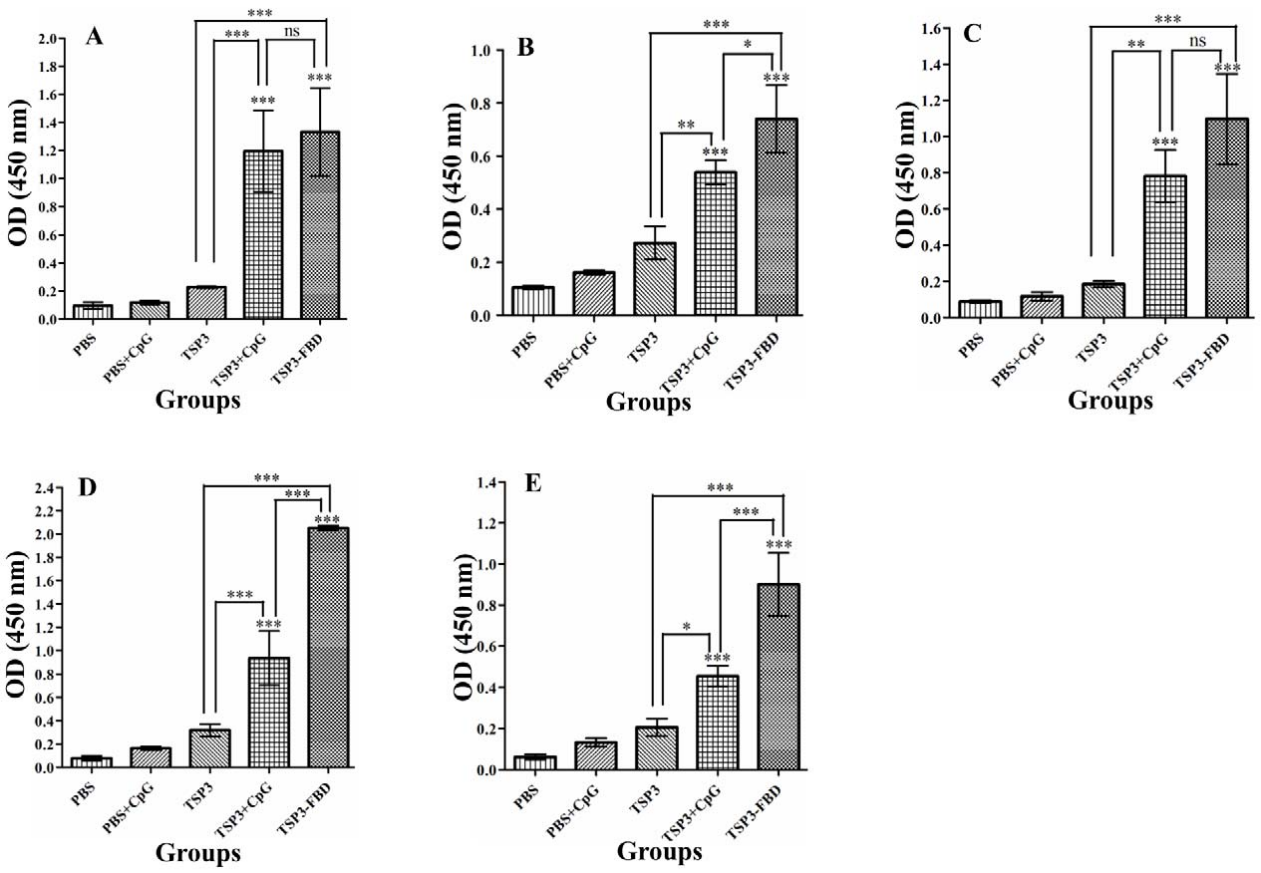
ELISA detection of local mucosal IgA responses. Local mucosal IgA antibody responses were detected in BALB/c mice immunized with rEm-TSP3+CpG or rEm-TSP3-FBP intranasally after the third immunization. Mice were sacrificed and nasal washes, liver extracts and intestinal washes were collected for measurement of nasal IgA (A), intestinal IgA (B), liver IgA (C), lung IgA (D) and spleen IgA (E) antibody responses. Significant differences between the vaccinated groups and the PBS control group are denoted by an asterisk over the bar. The S.D. is indicated by vertical lines. Significant differences between any of two groups are denoted by an asterisk over the line connecting them. n = 3 per group; *p<0.05 (significant); **p<0.01 (very significant); ***p<0.001 (extremely significant).

Very strong IgA responses (*p*<0.001) were detected in the nasal cavity samples from both the rEm-TSP3+CpG and rEm-TSP3-FBP groups (*p*<0.001), with no significant difference between them (Figure 5A). Significant IgA responses were detected in intestinal samples (*p*<0.001) from both groups, with the rEm-TSP3-FBP group being significantly higher (*p*<0.05) (Figure 5B). High liver IgA antibody levels were also detected in both groups (*p*<0.001), with no significant difference between them (Figure 5C). Significantly stronger IgA antibody responses were found in lung and spleen of both rEm-TSP3+CpG and rEm-TSP3-FBP groups (*p*<0.001). There was a clear difference between the groups, with the latter being extremely higher (Figure 5D,5E) (*p*<0.001). No significant IgA antibody responses were detected in any tissues of other control groups (Figure 5). Figure S1 shows the potential mechanism whereby Em-TSP3 induces strong mucosal antibody responses enhanced by fused FBP of *M. avium* FAP.

## Discussion

We have previously shown that i.n. vaccination of rEm-TSP3 with CpG ODN adjuvant induces strong systemic and local antibody responses with a >60% reduction in cyst lesion number reduction (CLNR) in the liver of BABL/c mice [13]. CpG DNA contains unmethylated CpG motifs (often given in the form of synthetic oligodeoxynucleotides (CpG ODN)), which contributes to its adjuvant activities by stimulating B cells [25] and activating DCs [26]. As a novel adjuvant, CpG OND induces Th1-like responses [27], [28]. CpG ODN has recently been shown to act as potent adjuvants for vaccines delivered by i.n. inhalation [29]–[31]. However, in our previous study it didn't induce satisfied intestinal IgA production [13]. Because the nature infection of AE is closely associated to gastrointestinal tract, intestinal IgA is thought to be the first lines of defences against early infection by *E. multilocularis* [32]. We evaluated the efficiency of potent molecules such as FAPs to act as an adjuvant and enhanced mucosal IgA antibody responses.

FAPs are a family of fibronectin-binding proteins that are present in several species of bacteria [22]. The attachment and internalization of *Mycobacterium* by epithelial cells were shown to be dependent on the interaction between FAPs and fibronectin [19], [21], [23], [33]. Moreover, after targeting and invasion of host M cells by *Mycobacterium* by the formation of a fibronectin bridge between *Mycobacterium* FAP and integrins on host M cells [20], FAP modulates adaptive immune responses by inducing maturation and activation of DCs, driving a predominantly Th1 polarization [34].

We are the first to create a vector containing the fusion form of *M. avium paratuberculosis* FBP and Em-TSP3. Em-TSP3 and FBP were linked with a GSGGSG linker, which is a commonly used flexible peptide in biofunctional fusions [35]. Hydrophobicity analysis showed that there was no significant change in solubility between the unfused and fused forms of Em-TSP3. Three-dimensional structure prediction of Em-TSP3 suggested that the exposure of the C-terminal linked with short FBP might improve the attachment of fusion protein to epithelial M cells and trigger DC activation [20].

*Echinococcus* metacestodes form a laminated layer which protects them from host immune attack after the infection established on the liver of intermediated host. Therefore, it may be more feasible to kill *Echinococcus* oncospheres in the early stage of infection in the intestine and blood before they develop into metacestode. As was suggested that antibodies form a critical part of the immune response against taeniid metacestodes, with IgG1, IgG2α, IgG2β and IgE playing a major role in oncosphere killing, although the involvement of other mechanisms should not be ruled out [36].

The tendency of Th1 or Th2 cell immune responses were assessed via detecting the IgG1/IgG2α ratio after the second and the third immunization. Immunization with rEm-TSP3-FBP resulted in an increased IgG1/IgG2α ratio (a tendency towards Th2), while rEm-TSP3+CpG showed an early Th1-dominated response that shifted towards a Th2 response later. T cells regulate Ig isotype switching on the basis of their ability to secrete cytokines. In mice, IL-4 (inducing IgG1 and IgE), IFN-γ (inducing IgG2α and IgG3) and TGF-β (inducing IgA and IgG2b) are the most important cytokines involved in Ig isotype switching [37], [38]. It is suggested that the Th1-polarized cytokine response plays an important role in killing *Echinococcus* metacestodes during the initial stage of development in liver. The response shifts to a predominantly non-protective Th2 response during the chronic stage [36], [39]–[47]. Although FAP was previously shown to induce a Th1-like response [34], in our study, the fusion of FBP to Em-TSP3 induced a predominantly Th2 response. Studies indicate that *E. granulosus* has developed strategies for immune evasion using molecules such as antigen B, whereby DCs differentiation is impaired, resulting in polarization towards a Th2 cell response [48]. Tetraspanin of *Schistosoma* was also shown to serve as an important molecule in immune evasion by masking their nonself status [49], [50]. Based on these and our recent studies, we believe that although FBP facilitated targeting of M cells by the fusion protein to stimulate stronger mucosal immune responses, it also caused tetraspanins to impair DCs functions [48]. This is the most likely explanation for the failure of rEm-TSP3-FBP to induce a high IgG2α response. We speculated that Em-TSP3 might be one of the most important molecules for regulating host immune responses by *Echinococcus* metacestode to benefit their long-term survival in their intermediate host [13].

Mucosal IgA responses were also detected by ELISA. Remarkably, immunization with rEm-TSP3-FBP evoked significantly strong IgA antibody responses in intestine, lung and spleen compared to that by rEm-TSP3+CpG. Research on mucosal immunological responses over the past decades has suggested that mucosal IgA plays a crucial role by neutralizing parasite ES (excretory-secretory) products. This leads to attenuation of the parasite-host interaction and interferes with parasite feeding and survival [51]–[53], inducing eosinophil degranulation [54] and tolerance induction to the parasite [32]. Secretory (intestinal) IgA is thought to be one of the first lines of defences against infections by parasites such as *Giardia* [55], *Trichinella* [56] and *Echinococcus* [32]. Moreover, in this study, higher liver IgA response was induced by rEm-TSP3-FBP compared to rEm-TSP3+CpG (*p*<0.05), which is thought to be one of the important immune-associated factors during the chronic stage of *Echinococcus* metacestode infection [39], [57], [58].

I.n. administration of the Em-TSP3 fused with FBP (rEm-TSP3-FBP) induced both systemic and local antibody responses, indicating that this is a novel, prospective model for the development of an efficient, non-toxic human vaccine. Since both the CpG and FBP did not induce the expected Th1 response against *Echinococcus* metacestode, we speculated that the early systemic (IgG) and mucosal (IgA) antibody responses are crucial for oncosphere killing and thus provided protection in our experimental model [13], [22]. It appears to be very difficult to exclude metacestodes completely once infection is established in the host liver, although the liver IgA may play an important role in anti-echinococcosis [39], [57], [58].

We chosen an i.n. administration route in this study because it is the most appropriate for inducing the full range of local immune responses (so-called ‘common mucosal immune system’) [59]. However, it is notable that although both the CpG and FBP provoked strong intranasal IgA responses, there was a significant difference in intestinal IgA production. It is clear that FBP is more efficient to induce IgA production at certain remote sites, e.g., intestine, lung and spleen. We noticed a swallowing behavior during the i.n. administration of Ags in all BALB/c mice which means antigens were partially administrated orally. Thus, FBP might be more efficient than CpG if orally administered. This should be confirmed by performing an oral vaccination experiment for further evaluation of their vaccine efficacy. Another important consideration for developing vaccines against *E. multilocularis* established its infection in the liver is to find a more efficient adjuvant that is able to offset the undesired effect of proteins like antigen B and tetraspanin which is believed to cause a shift from protective Th1 response to non-protective Th2 response [13], [48], or fuse FAP with other protective Ags.

## Supporting Information

Figure S1 **Schematic representation of mucosal anti-rEm-TSP3 specific IgA production against** ***Echinococcus*** **oncospheres enhanced by rEm-TSP3-FBP.** FBP of Em-TSP3-FBP (**T–F**) facilities binding of fusion protein to fibronectin (**F**) of microfold cells (**M**), a subepithelial dome rich in dendritic cells (**DC**), B cells (**B**) and plasma cells (**P**). After Em-TSP3-FBP is transported into M cells, DCs take up and present it directly to B cells and T cells (**T**), which induces IgA (**I**) class-switching and differentiation *in situ*. Secreted IgA is transported across the epithelium (**E**), where it serves as a first line of defences against *Echinococcus* oncospheres (**O**). A blue arrow indicates the enhanced binding of fusion protein to fibronectin of M cells.
(TIF)

Table S1 **List of Genbank accession numbers for the genes referred to in the text.**
(DOC)
