# Supplementary figures and images for: Mucosal Adjuvanticity of Fibronectin-Binding Peptide (FBP) Fused with Echinococcus multilocularis Tetraspanin 3: Systemic and Local Antibody Responses
Source: PLoS Negl Trop Dis. 2012 Sep 27;6(9):e1842. doi: 10.1371/journal.pntd.0001842 (PMC3459843; doi:10.1371/journal.pntd.0001842)

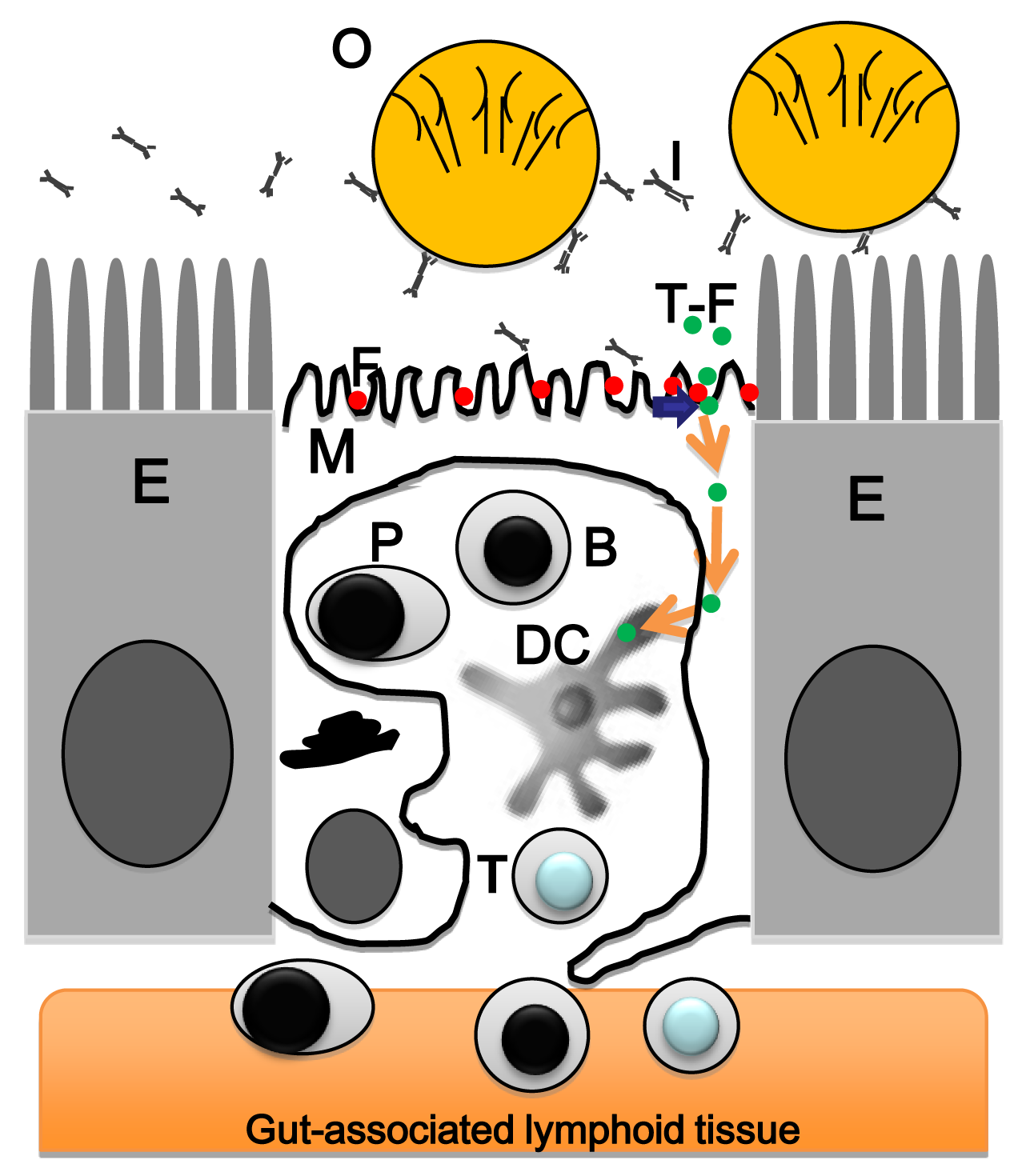

Supplement: Figure S1 — Schematic representation of mucosal anti-rEm-TSP3 specific IgA production against Echinococcus oncospheres enhanced by rEm-TSP3-FBP. FBP of Em-TSP3-FBP (T–F) facilities binding of fusion protein to fibronectin (F) of microfold cells (M), a subepithelial dome rich in dendritic cells (DC), B cells (B) and plasma cells (P). After Em-TSP3-FBP is transported into M cells, DCs take up and present it directly to B cells and T cells (T), which induces IgA (I) class-switching and differentiation in situ. Secreted IgA is transported across the epithelium (E), where it serves as a first line of defences against Echinococcus oncospheres (O). A blue arrow indicates the enhanced binding of fusion protein to fibronectin of M cells. (TIF) [file pntd.0001842.s001.tif]
