# Supplementary material for: Mucosal Adjuvanticity of Fibronectin-Binding Peptide (FBP) Fused with Echinococcus multilocularis Tetraspanin 3: Systemic and Local Antibody Responses
Source: PLoS Negl Trop Dis. 2012 Sep 27;6(9):e1842. doi: 10.1371/journal.pntd.0001842 (PMC3459843; doi:10.1371/journal.pntd.0001842)
Supplement: Table S1 — List of Genbank accession numbers for the genes referred to in the text. (DOC) [file pntd.0001842.s002.doc]

**Table S1. List of Genbank accession numbers for the genes referred to in the text**

| **Gene** | **Parasite** | **Accession number** | **Reference** |
| --- | --- | --- | --- |
| **SfbI** | *Streptococcus pyogenes* | X67947 | 14,15,16,18 |
| **FAP** | *Mycobacterium avium* | U53585 | 21 |
| **FAP** | *Mycobacterium leprae* | L01095 | 33 |
| **Em-TSP1** | *Echinococcus multilocularis* | FJ384717.1 | 23 |
| **Em-TSP2** | *E. multilocularis* | FJ384718.1 | 23 |
| **Em-TSP3** | *E. multilocularis* | FJ384719.1 | 23 |
| **Em-TSP4** | *E. multilocularis* | FJ384720.1 | 23 |
| **Em-TSP5** | *E. multilocularis* | FJ384721.1 | 23 |
| **Em-TSP6** | *E. multilocularis* | FJ384722.1 | 23 |
| **Em-TSP7** | *E. multilocularis* | FJ384716.1 | 23 |
| **EgAgB** | *E. granulosus* | M36774 | 48 |
| **Sm-TSP-1** | *Schistosoma mansoni* | AF521093 | 49 |
| **Sm-TSP-2** | *S. mansoni* | AF521091 | 49 |
